# Risks from mercury in anadromous fish collected from Penobscot River, Maine

**DOI:** 10.1016/j.scitotenv.2021.146691

**Published:** 2021-03-29

**Authors:** Lisa Jo Melnyk, John Lin, Daniel H. Kusnierz, Katherine Pugh, James T. Durant, Rene J. Suarez-Soto, Raghuraman Venkatapathy, Devi Sundaravadivelu, Anthony Morris, James M. Lazorchak, Gary Perlman, Michael A. Stover

**Affiliations:** a U.S. Environmental Protection Agency (EPA), Office of Research and Development, 26 W. Martin Luther King Drive, Cincinnati, OH 45268, United States of America; b Penobscot Indian Nation, Department of Natural Resources, 27 Wabanaki Way, Indian Island, ME 04468, United States of America; c EPA, Region 4, 61 Forsyth Street SW, Atlanta, GA 30303, United States of America; d Agency for Toxic Substances and Disease Registry, 4770 Buford Highway, Atlanta, GA 30341, United States of America; e Pegasus Technical Services, Inc., 26 W. Martin Luther King Drive, Cincinnati, OH 45268, United States of America; f Agency for Toxic Substances and Disease Registry, 5 Post Office Square, Suite 1010, Boston, MA 02109, United States of America; g US Public Health Service on detail to EPA, Region 1, 5 Post Office Square, Suite 100, Boston, MA 02109, United States of America

**Keywords:** Anadromous fish, Mercury, Consumption, Dose, One Health approach

## Abstract

Levels of total mercury were measured in tissue of six species of migratory fish (alewife, American shad, blueback herring, rainbow smelt, striped bass, and sea lamprey), and in roe of American shad for two consecutive years collected from the Penobscot River or its estuary. The resultant mercury levels were compared to reference doses as established in the U.S. Environmental Protection Agency (EPA) Integrated Risk Information System and wildlife values. Mercury concentrations ranged from 4 μg/kg ww in roe to 1040 μg/kg ww in sea lamprey. Sea lamprey contained the highest amounts of mercury for both seasons of sampling. Current health advisories are set at sufficient levels to protect fishers from harmful consumption of the fish for mercury alone, except for sea lamprey. Based upon published wildlife values for mink, otter, and eagle, consumption of rainbow smelt, striped bass, or sea lamprey poses a risk to mink; striped bass and sea lamprey to otter; and sea lamprey to eagle. For future consideration, the resultant data may serve as a reference point for both human health and wildlife risk assessments for the consumption of anadromous fish. U.S. EPA works with federally recognized Tribes across the nation greatly impacted by restrictions on sustenance fishing, to develop culturally sensitive risk assessments.

## Graphical Abstract

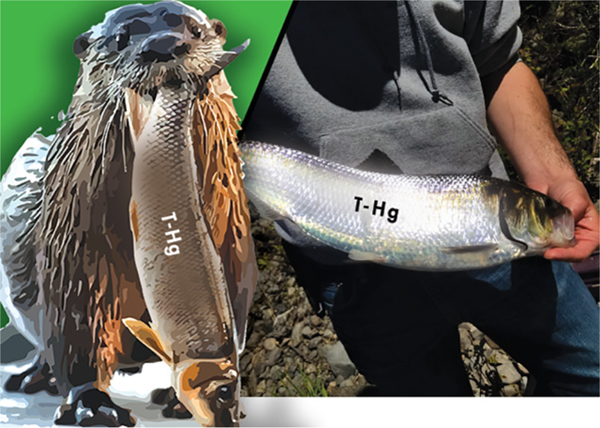

## 1. Introduction

Mercury contamination has been a long-standing environmental issue. Removal of mercury contamination from the environment has been challenging due to the ubiquitous nature of the metal and continuing global sources of mercury to the atmosphere. In the absence of a comprehensive method of mercury removal, the scientific community has focused on monitoring mercury levels in the environment, humans, and wildlife to establish baseline data, measure trends, and develop human consumption advisories. Mercury has been studied for many years (Wiener et al., 2012; Jenssen et al., 2012; Rothenberg et al., 2016; Kim et al., 2016; Eagles-Smith et al., 2017; EPA, 2015) and has been shown to impact human health and the environment, predominantly occurring in the form of methylmercury in aquatic food webs (Wiener et al., 2012). Health impacts to humans are well documented (cardiovascular and respiratory defects (Li and Tse, 2014); neurodevelopment (Rothenberg et al., 2016; Grandjean and Herz, 2011); and, to some extent, obesity (Wang et al., 2018)). Wildlife is impacted as well. Driscoll et al., 2013 has provided a complete description of mercury sources for both humans and wildlife (Driscoll et al., 2013).

The concentration of mercury in fish has been demonstrated through studies in the Great Lakes area (Wiener et al., 2012; Grieb et al., 2019), Colorado lakes (Herrman et al., 2018), areas influenced by power plants (Reash et al., 2019), rural Alaska (Bridges et al., 2020), and in U.S. rivers and streams (Wathen et al., 2015). However, the fish in these studies are residential, i.e., stay within the waterbody. For anadromous fish, those that transverse between rivers and oceans, the amount of mercury in the tissue is not well known (Grieb et al., 2019), and may be lower, i.e., safer to consume.

In Maine, the Penobscot River had been dammed for the purpose of industry for nearly two hundred years, blocking passage of many species of migratory fish. Since 2014, removal of obstructing dams and other restoration efforts have allowed several anadromous fish species indigenous to the Penobscot River to return to historic spawning areas throughout the watershed. The river is home to the Penobscot Indian Nation (PIN) and a source of sustenance fishing to the tribe. Anadromous fish that were once plentiful and a major component of the traditional diet of tribal members are now once again available to sustenance fishers. Elevated contaminant levels of inorganic and organic compounds in resident fish (EPA, 2015) have resulted in a recommendation of decreased fish consumption rates for the PIN (ATSDR, 2006; ATSDR, 2014) as compared to their traditional levels (Harper and Ranco, 2009). Little, if any, data are available on contaminant levels in anadromous fish returning to the Penobscot River. Because anadromous fish spend most of their lives in the ocean and estuaries, they are not likely to be exposed to the same local sources of pollution as resident fish and may be a healthier source of food to sustenance fishers.

The United States Environmental Protection Agency (EPA) conducted a study jointly with the Agency for Toxic Substances and Disease Registry (ATSDR) and the Penobscot Indian Nation to measure total mercury levels in anadromous fish that are only recently returning to the Penobscot River in Maine. The purpose of the study was to measure mercury levels in anadromous fish during two consecutive spawning seasons (2017 and 2018). As total mercury is used to determine fish advisories for the Penobscot River, the results can be used to evaluate the appropriateness of the current limits. The levels of mercury in the fish will be determined and used to evaluate potential risks to both humans and wildlife.

## 2. Methods

Anadromous fish species were collected during their upstream spawning migration in the Penobscot River in Maine. Tissue samples from the fillet portions of the fish typically consumed by the population were analyzed for total mercury. Using the mercury results, a culturally appropriate health evaluation was developed to determine the amount of risk Penobscot Nation members face when engaging in their legally protected right of sustenance fishing and their traditional cultural practices. The mercury levels were also converted to whole fish concentrations and compared to wildlife values for three animals to evaluate potential risk to these species. The fish species collected included alewife (*Alosa pseudoharengus*), American shad (*Alosa sapidissima*), blueback herring (*Alosa aestivalis*), rainbow smelt (*Osmerus mordax*), striped bass (*Morone saxatilis*), and sea lamprey (*Petromyzon marinus*). From the American shad, roe samples were collected as well.

### 2.1. Fish collection

**Fig. 1.**
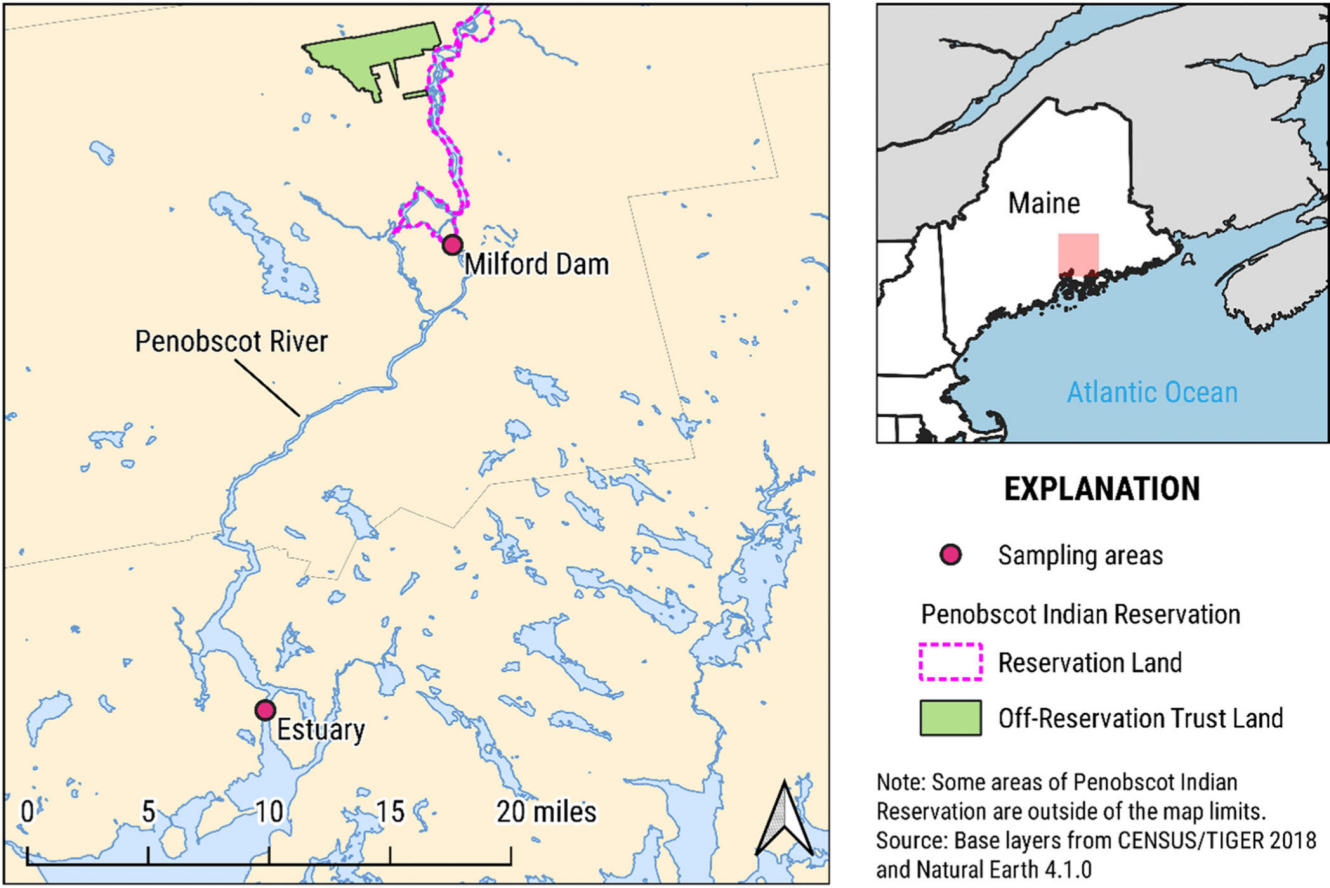
Map of sampling locations along Penobscot River.

Most of the fish were collected at the Milford Dam fish lift (operated as a run of river dam) located on the Penobscot River with assistance from the Maine Department of Marine Resources (Fig. 1). Some American shad and striped bass were collected by angling just downstream of the fish lift. Rainbow smelt were collected in 2017 in the estuary of the Penobscot River by National Oceanic and Atmospheric Administration trawls and in 2018 in spawning streams by night-time dip netting. These activities were performed or supervised by staff of the Penobscot Indian Nation Department of Natural Resources (PINDNR) in Maine.

### 2.2. Fish sample processing

Fish were kept alive in water or freshly killed on ice and transported to the PINDNR laboratory. Each fish was then measured for total length (tip of snout to tip of caudal fin), weighed, and filleted by removing the skinless tissue from both sides of the fish body from alewife, American shad, blueback herring, and striped bass. Rainbow smelt were processed as whole skin-on fish by removing the head and guts. Sea lamprey were gutted, severed into whole two to 4-in. pieces, skinned, and excised of fish tissue from the notochord. American shad roe were removed with sacs left intact. Fish tissue or roe from each individual was weighed, double wrapped in foil, labelled and frozen at minus 20 °C. Based on weight and length, each species of fish was separately combined, to create a composite of three to ten individual fish (both fillets constitute one fish). The fish sizes for a composite sample were within 75% by length of the largest fish and within 75% by length of each other. The composite sample was of sufficient mass to complete all of the analyses. Five or six composites were created for each fish species and roe. Fish tissue was composited to allow for an averaged concentration over a greater number of individual fish samples used to determine exposure potentials.

### 2.3. Fish analysis

Fish portions, fillets, and roe composite samples were shipped from the preparation laboratory to the EPA analysis laboratory (Cincinnati, Ohio). A total of seventy-five of these composite samples were shipped for analysis of total mercury. All samples were measured for moisture and lipid content. Details for sample receiving, shipping, and processing followed established procedures for the laboratory.

The entire contents of the fish composites were homogenized using a stainless-steel blender (Waring, Torrington CT), mixed to a fine paste of uniform color and texture. To determine moisture content, a one to five-gram aliquot of homogenized fish tissue was dried at 110 ± 5 °C using an oven that would maintain this temperature until the final dry weight did not vary by more than ±0.0005 g. The moisture content was determined by subtracting the final weight from the starting weight. For lipid content, 3 g of fish tissue homogenate were dried with diatomaceous earth for 1 h and then extracted with 1:1 (v/v) hexane:isopropanol (2 cycles each at 100 °C, 1500 psi, 5 min static time, 290 s purge time, and 140% flush volume) using an Accelerated Solvent Extraction (ASE 350) unit (Thermo Scientific Dionex, Waltham, MA). The extract was blown down to a final volume of 25 mL under the flow of nitrogen at 50 °C (Zymark TurboVap II, Hopkinton, MA). An aliquot of the extract was air dried in a fume hood, and then placed in a 50 °C oven for 90 min to complete drying. The lipid weight in the extract was calculated from the final weight subtracted from the starting weight and finally corrected for the mass of tissue extracted. A Laboratory Reagent Blank (LRB) with only hydromatrix and SRM1947 with a lipid content of 10.4 ± 0.5% was also analyzed in each extraction batch. The results were < 0.0005% and 10.3 ± 0.9 (n = 6), respectively.

Next, a 100-milligram aliquot of the homogenized fish tissue was used for mercury analysis. Three replicates of each composite were analyzed for mercury.

Total mercury in fish tissue and roe was analyzed using the Direct Mercury Analyzer–80 (DMA-80) with interfaced analytical balance (Milestone Inc., Monroe, CT). Thermal decomposition and amalgamation followed by atomic absorption spectrophotometry without sample pretreatment or preparation was performed according to the protocols of U.S. EPA SW-846 Method 7473 (EPA, 2007). Calibration standards and known additions of mercury were made with a solution traceable to the U.S. National Institute of Standards and Technology (U.S. NIST) from SPEX CertiPrep (Metuchen, NJ). The accuracy of the standard was verified against another certified source from ACROS Organics. The method was validated by analysis of a standard reference material (SRM1947–Lake Michigan Fish Tissue) and fish tissues fortified with known quantities of mercury.

Amounts of total mercury in reference materials were within certified ranges and the average recoveries for the fortified samples were greater than 90% (*n* = 16). Duplicate samples were also analyzed per sequence and the relative percent difference averaged less than 5% (*n* = 22). The limit of detection (0.013 ng/kg) for the DMA-80 was evaluated based on the procedure detailed in 40CFR 136, Appendix B, Revision 1.11. The sample concentrations on average were at least 500 times higher than the detection limit.

### 2.4. Statistical analysis

R version 3.6.1 (R Core Team, 2019) was used for data analysis and visualization. Goodness of fit tests were performed using EnvStats (Millard, 2013) implementation of the Shapiro-Wilk test for normality. Because technical replicates were analyzed from each composite sample, the survey package version 3.35–1 (Lumley, 2004) was used to perform summary statistics (mean, 95% confidence intervals using delta method) and to perform pairwise comparisons across size and year strata using two-sided *t*-tests. Tukey’s multiple comparisons were used for the effect of species on total mercury (Hothorn et al., 2008). The use of the survey methods is to account for clustering using the complex design with cluster set to composite sample identifier. Linear regression was used to evaluate the interactive effect of species and percent lipids on log of mean mercury samples.

### 2.5. Human health evaluation

The concentration of total mercury in fish tissue was used to calculate the exposure dose (Eq. (1)), which was compared to the Reference Dose established by EPA to obtain the hazard quotient (HQ) (Eq. (2)). A HQ of less than 1 indicates safe consumption at the levels established for the community consuming fish from the Penobscot River.

(1) D=(C×IR×EF)/BW

where: D = exposure dose (μg/kg/day), C = contaminant concentration (μg/kg), IR = ingestion rate (kg/day), EF = exposure factor (unitless), BW = body weight (kg).

(2) HQ=D/RfD

where: HQ = hazard quotient, D = exposure dose (μg/kg/day), RfD = reference dose (μg/kg/day).

The ingestion rate used for the community was 40 g/day (10 oz/week) for adult tribal members, which is the highest rate of consumption from current PIN advisories (https://www.penobscotnation.org/images/natural-resources/Documents/PIN-Fish-Brochure-Final-Draft-01162018R.pdf). The Exposure Factor for non-cancer is 1. The BW of 80 kg was used to be more realistic of current averages and to be consistent with EPA’s Exposure Factors Handbook (EPA, https://www.epa.gov/expobox/about-exposure-factors-handbook). It was assumed that all the mercury detected in fish and roe samples was in the form of methylmercury (Bloom, 1992; Jones and Slotten, 1996; Wageman et al., 1997; Katner et al., 2010; Wentz et al., 2014). This would ensure the worst-case scenario and be the most protective for safe consumption. Therefore, the reference dose for methylmercury of 0.1 μg/kg BW/day was used as provided by the Integrated Risk Information System (EPA, https://cfpub.epa.gov/ncea/iris2/chemiclLanding.cfm?substance_nmbr=73).

### 2.6. Wildlife assessment

Human consumption of these fish species does not constitute a complete picture of the possible impact of mercury-contaminated fish. Wildlife also have access to and consume these fish with potential exposure to mercury (Walters et al., 2010; Lazorchak et al., 2003; Peterson et al., 2004). However, wildlife do not consume merely the fillets or portions as humans typically do, but rather the entire fish, so the results of the composite fish samples were converted to an equivalent whole fish concentration (Wathen et al., 2015). The wildlife values for mink, otter, and eagle were compared to concentrations converted from fillet and fish portion values to equivalent whole-fish values using a factor of 0.62 (Wathen et al., 2015) to determine if any wildlife values were exceeded, thereby constituting a harmful potential exposure to mercury:

(3) $$\text{ Whole fish concentration }={\text{C}}_{\text{fillet }}\times 0.62$$

where: C = contaminant concentration (μg/kg) in fillet or fish portions, 0.62 = conversion factor.

## 3. Results

### 3.1. Levels of total mercury in anadromous fish samples

Total mercury concentrations, moisture, and lipid contents are summarized in Table 1 for both 2017 and 2018 samples and ranged from 4 μg/kg ww in roe samples to 1040 μg/kg ww in sea lamprey with the fish tissue moisture between 70 and 80%. In 2018, the sizes of rainbow smelt varied more than the compositing criteria would allow, so separate samples designated large and small were created. The raw wet weight concentrations varied by fish species. The number of individual fish within a composite differed because of size and availability. The small sized fish required greater numbers of individuals to create enough sample to complete all analyses.; therefore, rainbow smelt which were small needed 10 individuals for a composite, whereas, sea lamprey, a large fish, required only 3. When the quantity of fish was high, an additional composite was made. Thus, six composites were created in 2018 for American shad fillet and roe. The compositing scheme allowed for a greater number of individual fish to be analyzed.

Alewife (combined mean of 90.8 μg/kg), rainbow smelt (combined mean of 120 μg/kg), striped bass (combined mean of 166 μg/kg), and sea lamprey (combined mean of 628 μg/kg) were consistent in terms of mercury content from one year to the next. American shad (combined mean of 72 μg/kg), roe (combined mean of 8.62 μg/kg), and blueback herring (combined mean of 51.5 μg/kg) increased in mercury levels from 2017 to 2018 (*P* < 0.05). Levels of mercury in American shad roe from 2017 were not normally distributed (*P* < 0.05) using Shapiro-Wilk test, indicating the variability within these samples was high. For the rainbow smelt in 2018, the large sized fish appear to have slightly more mercury than the smaller sized, but the difference was not significant (*P* = 0.06). Overall, sea lamprey contained the highest levels of mercury which were approximately 4-fold greater than levels in the next highest species (striped bass, rainbow smelt).

Lipid content ranged between 0.6% in striped bass from 2018 to 7.8% in roe samples from 2017. There were no significant effects of percent lipid on log of mercury concentrations, except for American shad roe, where a 1% increase in lipids was associated with a 26% decrease in mercury (*p* < 0.05) and rainbow smelt, all sizes combined, where a 1% increase in lipid was associated with a 110% increase in mercury (p < 0.05).

Taking into consideration the trophic levels of these fish species, where striped bass and sea lamprey are in level 4; American shad, alewife and blueback herring are in level 3; and rainbow smelt could be in either level 3 or 4 (EPA, https://www.epa.gov/ecobox/epa-ecobox-tools-exposure-pathways-food-chains; EPA, 2003), the mercury concentrations follow that the higher the trophic level, the greater the mercury amounts were detected or bioaccumulated. Using the Tukey multiple comparison test, a comparison of the mercury concentrations based on trophic levels resulted in sea lamprey being significantly higher (p < 0.05) than all other species. Considering their parasitic nature, it is assumed that mercury transferred from the fish on which they were feeding in addition to environmental impacts. Striped bass and rainbow smelt contained similar amounts of mercury (*p* = 0.18). Mercury in alewife was significantly lower (p < 0.05) than in striped bass but did not differ from rainbow smelt (*p* = 0.19). American shad, blueback herring, and American shad roe contained significantly different (p < 0.05) amounts of mercury from all other fish species tested.

### 3.2. Human health evaluation

For this study, exposure scenarios to mercury were determined from consumption of anadromous fish and whether the resultant level could potentially cause health problems. As mercury is a concern to the Penobscot Nation community, the results can be used by the Penobscot Nation to inform revisions to recommendations to the health advisories currently in existence, if warranted.

Table 2 summarizes the ranges of dose calculations for the health evaluation for all the composite samples, separated by year of collection. Included is the calculated HQ used to determine whether the levels of mercury in the fish allow for safe consumption (ATSDR, 2020). Sea lamprey contained levels of mercury that consistently had a HQ >1 for both years of sampling. For 2017, two composite samples of striped bass contained mercury at levels that resulted in a HQ greater than 1, and the confidence interval for mean HQ covering 1. These values are shaded in the table. Using a fish consumption rate of 10 oz/week (approximately 40 g/day) for all of the fish species for adults, and taking into account the resultant mercury levels in the analyzed 2017 and 2018 fish tissue samples, consumption of alewife, American shad fillet and roe, blueback herring, and rainbow smelt is deemed safe at the suggested consumption rate. A recommendation to the community was made to refrain from consuming sea lamprey.

### 3.3. Impact on wildlife species

Converted mercury levels from fillets to whole fish are summarized in Table 3. Various species of wildlife are potentially susceptible to mercury which can threaten their survival. The shaded areas indicate that the level of mercury in the whole-fish composite samples for certain fish species exceeded the wildlife value for mink at 70 μg/kg, otter at 100 μg/kg, or eagle at 160 μg/kg (Wathen et al., 2015). These levels of mercury potentially place minks at risk when consuming rainbow smelt, striped bass, or sea lamprey. Otters are potentially at risk when consuming striped bass and sea lamprey. Eagles are potentially at risk when consuming sea lamprey.

## 4. Discussion

American Indian tribes have unique traditional cultural practices that are often not adequately protected by using default EPA fish consumption rates. A previous investigation by EPA concluded that the current sustenance fishing practices of the Penobscot Indian Nation are threatened due to unsafe levels of mercury found in the tissue of resident fish species (EPA, 2015). Resident fish, snapping turtles, wood ducks and some plants were measured and found to contain 290–708 μg/kg (wet weight), 963 μg/kg, 49 μg/kg, and nondetectable levels of mercury, respectively, which has resulted in issuance of health advisories based on total mercury to limit consumption of resident fish and other species (https://www.penobscotnation.org/images/natural-resources/Documents/PIN-Fish-Brochure-Final-Draft-01162018R.pdf).

Recent river restoration efforts, including dam removals, have resulted in the abundant return of several anadromous fish species to the Penobscot River where they have been missing for the past 200 plus years. These returning fish may serve to restore a major component of the traditional diet of the Penobscot Indian Nation. With the resident fish containing between 290 and 708 μg/kg of mercury, some anadromous fish offer an alternative food source with potentially less mercury toxicity.

This project found that the average levels of mercury in the anadromous fish generally were below the levels of resident fish and, therefore, fall within the fish advisories already established by the Penobscot Indian Nation. Based on mercury alone, existing advisories by the Penobscot Nation are adequately protective, except for sea lamprey. It is recommended that sea lamprey not be consumed at any ingestion rate at this time. The advisories assist the Penobscot Nation in reducing the risk for tribal members from being exposed to toxic levels of mercury and provide information to assess the sustainability of a traditional Penobscot subsistence diet during current times. However, caution needs to be taken as mercury is not the only contaminant present in these fish species (ATSDR, 2020). Other chemicals determined in the fish render them unsafe to consume at any ingestion rate.

There are some notable limitations and sensitivities with the health evaluation. With HQs near 1, attention should be paid to length of striped bass as age affects mercury levels in these species (Gochfeld et al., 2012; Cizdziel et al., 2002). The assessment of human dose is directly proportional to intake rates, which was assumed to be the highest consumption rate under the Penobscot Indian Nation’s current fish advisory guidelines. However, subsistence-traditional lifeways intake values, which are higher than guideline limits should be considered. The Wabanaki Traditional Cultural Lifeways Exposure Scenario (Harper and Ranco, 2009) is a peer-reviewed document to inform decisions regarding tribal designated uses when reviewing or approving water quality standards, suggests a freshwater fish intake of 286 g per day for adults and 143 g per day for children. The Wabanaki Traditional Cultural Lifeways Exposure Scenario provides suggested numerical representation of uses and exposure pathways of traditional subsistence lifeways of tribal members who would otherwise fully use natural resources and traditional lifeways if not prevented by present-day environmental conditions. Therefore, the hazard quotients could be approximately seven times higher under this consumption pattern. The approaches used to make public health determinations in this publication are similar to EPA risk assessment methods. Furthermore, other contaminants detected in these fish species may drive public health decisions beyond what mercury alone would indicate.

In addition to the health of the Penobscot Indian Nation’s members, wildlife concerns are also important. Levels of mercury found in several of the fish species analyzed pose a potential risk for wildlife that are important to the Penobscot Indian Nation, such as the eagle. Rainbow smelt, striped bass, and sea lamprey all contained levels of mercury that exceeded safe wildlife values, although the exact source(s) of mercury in these fish is unknown and beyond the scope of this study. Additional research into the source(s) of mercury in these fish would be helpful in reducing the risks to wildlife.

This study links the Penobscot Nation to science, policy, and regulatory decision making within Indian Country. The data may serve to inform the review or development of water quality standards that are protective of tribal practices. Since EPA has been working with several tribes nationally to develop culturally sensitive risk assessments, the results of the study may be transferable to tribal nations across the country.

The results of this study clearly indicate the impact that mercury contamination can have on marine life as analyzed in anadromous fish. As fish are a major source of omega 3 fatty acids which are high in nutritional value, every effort should be made to decrease the source of mercury, thereby, decreasing exposure to both humans and wildlife.

## Figures and Tables

**Table 1:** Concentration of mercury in anadromous fish collected from Penobscot River, micrograms per kilogram [wet weight].

Species	Year	Composite samples	Number of fish per composite	Percent moisture	Percent lipids	Composite range (3 replicates/composite averaged)	Mean (95% CI)
Alewife	2017	5	6	76.3	1.9	84.1–92.6	89.4 (86.3–92.6)
	2018	5	7	77.1	2.6	87.4–99.9	92.3 (87.9–96.7)
American shad fillet	2017	5	7	70.6	5.1	59.4–73.2	65.3 (59.9–70.8)
	2018	6	5	75.1	3.4	65.9–86.3	78.7 (71.7–85.7)^b^
American shad roe	2017	5	3	75.1	7.8	4.05–10.3	5.55 (2.97–8.12)^a^
	2018	6	3	73.3	3.6	6.93–18.4	11.7 (7.82–15.6)^b^
Blueback herring	2017	5	5	76.2	3.4	36.6–44.3	40.8 (37.8–43.7)
	2018	5	6	76.5	2.8	58–64.8	62.2 (59.6–64.9)^b^
Rainbow smelt	2017	5	3	81.6	1.2	57.1–182	99 (46.9–151)
Rainbow smelt, large	2018	3	10	79.8	1.6	137–148	143 (137–149)
Rainbow smelt, small	2018	3	10	80.0	1.4	97.3–135	118 (96.7–139)
Striped bass	2017	5	5	77.6	1.4	131–245	187 (144–229)
	2018	5	5	79.9	0.6	129–174	145 (129–162)
Sea lamprey	2017	6	3	76.4	5.0	361–880	620 (442–798)
	2018	6	3	78.0	3.3	387–1040	637 (430–843)

a Shapiro-Wilk test *P* < 0.05 for null hypothesis of normality.

b Increased mercury from 2017 to 2018.

**Table 2:** Health evaluation dose calculation, μg/kg/day.

Species	Year	Composite samples	Dose range (average 3 replicates)	Average HQ^a^
Alewife	2017	5	0.042 – 0.046	0.45
	2018	5	0.044 – 0.05	0.46
American shad fillet	2017	5	0.03 – 0.037	0.33
	2018	6	0.033 – 0.043	0.39
American shad roe	2017	5	0.002 – 0.0052	0.028
	2018	6	0.0035 – 0.0092	0.059
Blueback herring	2017	5	0.018 – 0.022	0.20
	2018	5	0.029 – 0.032	0.31
Rainbow smelt	2017	5	0.029 – 0.091	0.50
Rainbow smelt, large	2018	3	0.068 – 0.074	0.72
Rainbow smelt, small	2018	3	0.049 –0.068	0.59
Striped bass	2017	5	0.066 – 0.12	0.93
	2018	5	0.064 – 0.087	0.73
Sea lamprey	2017	6	0.18 – 0.44	3.10
	2018	6	0.19 – 0.52	3.20

a HQ - hazard quotient.

**Table 3:** Mercury concentration conversion to whole fish^a^ for wildlife assessment, micrograms per kilogram [wet weight].

Species	Year	Composite samples	Concentraon range in fillet	Converted average concentraon in whole fish (average of 3 replicates/composite)
Alewife	2017	5	52.1 – 57.4	55.4
	2018	5	54.2 – 61.9	57.2
American shad fillet	2017	5	36.8 – 45.4	40.5
	2018	6	40.8 – 53.5	48.8
Blueback herring	2017	5	22.7 – 27.5	25.3
	2018	5	36.0 – 40.2	38.6
Rainbow smelt	2017	5	35.4 – 113	61.4
Rainbow smelt, large	2018	3	85.0 – 91.7	88.8
Rainbow smelt, small	2018	3	60.3 – 83.4	73.1
Striped bass	2017	5	81.5 – 152	116
	2018	5	79.8 – 108	90.1
Sea lamprey	2017	6	224 – 545	404
	2018	6	240 – 642	395

Shading indicates exceedance of mink (70 μg/kg) and/or otter (100 μg/kg) and/or eagle (160 μg/kg) wildlife risk values.

a Whole body calculation based on 0.62 times fillet concentration.

## HIGHLIGHTS

- A One Health approach was utilized.
- Included anadromous fish have been rarely studied.
- Both humans and animals are at potential risk from consuming anadromous fish.
- Sea lamprey contained the highest concentration of mercury.
- Current consumption advisories are adequate for mercury only, except for sea lamprey.

## References

[R1] Agency for Toxic Substances and Disease Registry (ATSDR). 2006. Health consultation Penobscot River basin located near Lincoln, Maine. Atlanta, GA: U.S. Department of Health and Human Services, Public Health Service, 6 2006. https://www.atsdr.cdc.gov/HAC/pha/PenobscotRiverBasin2006/PenobscotHC20060106.pdf (accessed June 9, 2020).

[R2] Agency for Toxic Substances and Disease Registry (ATSDR). Public health assessment review of sediment and biota samples: Penobscot River Penobscot Indian Nation, Maine. Atlanta, GA: U.S. Department of Health and Human Services, Public Health Service, 7 2014. https://www.atsdr.cdc.gov/HAC/pha/PenobscotRiver/Penobscot%20River%20PHA%20(final)%20_%2007-18-2014_508.pdf (accessed June 9, 2020).

[R3] Agency for Toxic Substances and Disease Registry (ATSDR). Health Consultation - Public Comment Version Review of Anadromous Fish: Penobscot River Penobscot Indian Nation Indian Island, Maine. Atlanta, GA: U.S. Department of Health and Human Services, Public Health Service, 10 2020. [accessed 2020 March 8]. Available from: https://www.atsdr.cdc.gov/hac/pha/PenobscotRiver/Penobscot_Indian_Nation_HC-PC-508.pdf.

[R4] Bloom NS, 1992. On the chemical form of mercury in edible fish and marine invertebrate tissue. Can. J. Fish. Aquat. Sci 49 (5), 1010–1017.

[R5] Bridges KN, Furind CG, Gerlach RF, 2020. Subsistence fish consumption in rural Alaska: using regional monitoring data to evaluate risk and bioavailability of dietary methylmercury. Sci. Total Environ 736, 139676. 32497892 10.1016/j.scitotenv.2020.139676

[R6] Cizdziel J, Hinners T, Pollard J, Heithmar E, Cross C, 2002. Mercury concentrations in fish from Lake Mead, USA, related to fish size, condition, trophic level, location, and consumption risk. Arch. Environ. Contam. Toxicol 43, 309–317. 10.1007/s00244-002-1191-6. 12202927

[R7] Driscoll CT, Mason RP, Chan HM, Jacob DJ, Pirrone N, 2013. Mercury as a global pollutant: sources pathways, and effects. Environm. Sci Technol 47, 4967–4983. 10.1021/es305071v PMC3701261 23590191

[R8] Eagles-Smith CL, Silbergeld EK, Basu N, Bustamante P, Diaz-Barriga F, Hopkins WA, Kidd KA, Nyland JF, 2017. Modulators of mercury risk to wildlife and humans in the context of rapid global change. Ambio 47 (2), 170–197. 10.1007/s13280-017-1011-x PMC5794686 29388128

[R9] Gochfeld M, Burger J, Jeitner C, Donio M, Pittfield T, 2012. Seasonal, locational and size variations in mercury and selenium levels in striped bass (Morone saxatilis) from New Jersey. Environ. Res 112, 8–19. 22226733 10.1016/j.envres.2011.12.007 PMC4193446

[R10] Grandjean P, Herz KT, 2011. Brain development and methylmercury: underestimation of neurotoxicity. Mt Sinai J. Med 78 (1), 107–118. 10.1002/msj.20228. 21259267 PMC3096460

[R11] Grieb TM, Fisher NS, Karimi R, Levin L, 2019. An assessment of temporal trends in mercury concentrations in fish. Ecotoxicology 29, 1739–1749. 10.1007/s10646-019-02112-3. 31583510

[R12] Harper B, Ranco D, 2009. Wabanaki Traditional Cultural Lifeways Exposure Scenario. https://www.epa.gov/tribal/wabanaki-traditional-cultural-lifeways-exposure-scenario. (Accessed 2 April 2020).

[R13] Herrman SJ, Nimmo DWR, Vanden Heuvel BD, Carsella JS, Kennedy CM, Rogers KB, Wood JS, Herrmann-Hoesing LM, 2018. Mercury and selenium in twelve cut-throat trout tissues for high-elevation Colorado Lakes, USA: intraspecific and interspecific comparisons. Trans. Am. Fish. Soc 147, 444–458.

[R14] Hothorn T, Bretz F, Westfall P, 2008. Simultaneous inference in general parametric models. Biom. J 50 (3), 346–363. 18481363 10.1002/bimj.200810425

[R15] Jenssen MTS, Brantsaeter AL, Haugen M, Meltzer HM, Larssen T, Kvalem HE, Birgisdottir BE, Thomassen Y, Ellingsen D, Alexander J, Knutsen HK, 2012. Dietary mercury exposure in a population with a wide range of fish consumption—self capture of fish and regional differences are important determinants of mercury in blood. Science of the Total Environment 439, 220–229. 10.1016/j.scitotenv.2012.09.024 23069934

[R16] Jones AB, Slotten DG, 1996. Mercury effects, sources, and control measures; a special study of the San Francisco estuary regional monitoring program. San Francisco Estruary Institute, Richmond, CA. https://www.sfei.org/sites/default/files/biblio_files/mercury.pdf.

[R17] Katner A; Sun M; Suffet M. An evaluation of mercury levels in Louisiana fish: trends and public health issues. Science of the Total Environment 2010, 408(23): 5707–5714. 10.1016/j.scitotenv.2010.08.021 20855108

[R18] Kim KH; Kabir E; Jahan SA A review on the distribution of Hg in the environment and its human health impacts. J. Hazard. Mater 2016, 306: 376–85. 26826963 10.1016/j.jhazmat.2015.11.031

[R19] Lazorchak JM, McCormick FH, Henry TR, Herlihy AT, 2003. Contamination of fish in streams of the mid-Atlantic region: an approach to regional indicator selection and wildlife assessment. Environ. Toxicol 22, 545–553. 12627641

[R20] Li WC, Tse HF, 2014. Health risk and significance of mercury in the environment. Environ. Sci. Pollut. Res 22, 192–201. 10.1007/s11356-014-3544-x 25220768

[R21] Lumley T, 2004. Analysis of complex survey samples. J. Stat. Softw 9 (1), 1–19.

[R22] Millard SP EnvStats: An R Package for Environmental Statistics. 2013. Springer, New York. ISBN 978-1-4614-8455-4.https://www.springer.com/us/book/9781461484554#otherversion=9781461484561. (accessed April 2, 2020).

[R23] Penobscot Indian Nation. n.d. Wild Foods Safety Series – Fish Available from: https://www.penobscotnation.org/images/natural-resources/Documents/PIN-Fish-Brochure-Final-Draft-01162018R.pdf (accessed June 17, 2020).

[R24] Peterson SA, Van Sickle J, Hughes RM, Schacher JA, Echols SF, 2004. A biopsy procedure for determining fillet and predicting whole-fish mercury concentration. Arch. Environ. Contam. Toxicol 48, 99–107. 10.1007/s00244-004-0260-4 15657811

[R25] R Core Team. R: A Language and Environment for Statistical Computing. R Foundation for Statistical Computing, Vienna, Austria. 2019. https://www.R-project.org/.(accessed April 2, 2020).

[R26] Reash RJ, Friedrich LA, Bock MJ, Halden NM, Palace VP, 2019. Selenium and mercury in freshwater fish muscle tissue and otoliths: a comparative analysis. Environ. Toxicol. Chem 38 (7), 1467–1475. 30934135 10.1002/etc.4432

[R27] Rothenberg SE, Yu X, Liu J, Biasini FJ, Hong C, Jiang X, Nong Y, Cheng Y, Korrick SA, 2016. Maternal methylmercury exposure through rice ingestion and offspring neurodevelopment: a prospective cohort study. Int. J. Hyg. Environ. Health 219, 832–842. 27503636 10.1016/j.ijheh.2016.07.014 PMC5086436

[R28] U. S. Environmental Protection Agency. Methodology for Deriving Ambient Water Quality Criteria for the Protection of Human Health (2000); EPA/822-R-03-030; Office of Water, 12 2003.

[R29] U. S. Environmental Protection Agency. EPA Method 7473: Mercury in Solids and Solutions by Thermal Decomposition Amalgamation and Atomic Absorption Spectrophotometry. U.S. EPA, Office of Research and Development. 2007.

[R30] U. S. Environmental Protection Agency. The Penobscot River and Environmental Contaminants: Assessment of Tribal Exposure Through Sustenance Lifeways; EPA/901-R-15-002; Office of Research and Development, U. S. EPA, Boston, MA. 2015.

[R31] U. S. Environmental Protection Agency. n.d.-a Exposure Factors Handbookhttps://www.epa.gov/expobox/about-exposure-factors-handbook (accessed May 18, 2020).

[R32] U. S. Environmental Protection Agency. n.d.-b Integrated Risk Information System (IRIS) for Methylmercury, U. S. EPA; https://cfpub.epa.gov/ncea/iris2/chemicalLanding.cfm?substance_nmbr=73 (accessed April 2, 2020).

[R33] U. S. Environmental Protection Agency. n.d.-c EPA EcoBox Tools by Exposure Pathways – Food Chains. https://www.epa.gov/ecobox/epa-ecobox-tools-exposure-pathways-food-chains. Last accessed 3/10/21.

[R34] Wageman R, Tregacz E, Hunt R, Boila G, 1997. Percent methylmercury and organic mercury in tissues of marine mammals and fish using different experimental and calculation methods. Environ. Toxicol. Chem 16, 1859–1866.

[R35] Walters DM, Blocksom KA, Lazorchak JM, Jicha T, Angradi TR, Bolgrien DW, 2010. Mercury contamination in fish in midcontinent great rivers of the United States: importance of species traits and environmental factors. Environ Sci Technol 44, 2947–2953. 20297812 10.1021/es903754d

[R36] Wang X; Mukerjee B; Park SK Associations of cumulative exposures to heavy metals mixtures with obesity and its comorbidities among US adults in NHANES. Environment International 2018, 121, Pt 1. 10.1016/j.envint.2018.09.035 PMC6268112 30316184

[R37] Wathen JB, Lazorchak JM, Olsen AR, Batt A, 2015. A national statistical survey assessment of mercury concentrations in fillets of fish collected in the U.S. EPA national rivers and streams assessment of the continental USA. Chemosphere 122, 52–61. 25434269 10.1016/j.chemosphere.2014.11.005

[R38] Wentz DA; Brigham ME; Chasar LC; Lutz MA; Krabbenhoft DP Mercury in the nation’s streams – levels, trends, and implications. Circular 1395. USGS Report 2014, 10.3133/cir1395.

[R39] Wiener JG, Evers DC, Gay DA, Morrison HA, Williams KA, 2012. Mercury contamination in the Laurentian Great Lakes region: introduction and overview. Environ. Pollut 161, 243–251. 22000118 10.1016/j.envpol.2011.08.051

